# A multidimensional Framework for Routine Outcome Measurement in Liaison Psychiatry (FROM-LP)^†^

**DOI:** 10.1192/pb.bp.115.051458

**Published:** 2016-08

**Authors:** Peter Trigwell, James Kustow

**Affiliations:** 1 Yorkshire Centre for Psychological Medicine, Leeds and York Partnership NHS Foundation Trust; 2 Barnet, Enfield and Haringey Mental Health NHS Trust, London

## Abstract

In the field of liaison psychiatry, as in all areas of healthcare, there is an essential need for well-organised and consistent collection of information on outcomes, from a range of perspectives. This special article introduces, and describes the development of, the multidimensional Framework for Routine Outcome Measurement in Liaison Psychiatry (FROM-LP). This was challenging owing to the variety of service settings and types of intervention which characterise liaison psychiatry. Similar challenges may be faced by other specialties and this, along with the direct relevance of much of the eventual content of the framework, will broaden the interest of this article.

This article introduces the Framework for Routine Outcome Measurement in Liaison Psychiatry (FROM-LP), of key importance for all clinicians working in the field of liaison psychiatry and psychological medicine. The framework is also of relevance to others working in mental health who need to introduce an organised approach to outcome measurement in their own field or service, as much of the content is directly transferable.

The FROM-LP has been published as a faculty report by the Royal College of Psychiatrists.^1^

## Background

Over the past few years, in common with many areas of practice within the National Health Service (NHS), there has been an increasing focus on outcome and performance measurement in liaison psychiatry services. Various options and approaches have been considered during that period, but this did not lead to the identification of an agreed way forward. This became particularly important owing to the fact that, although there is mounting evidence for the economic benefit of liaison psychiatry services,^2^ there is a relative lack of information and evidence relating to clinical and other outcomes.^3^

The wider context also includes an increasing emphasis across the NHS on the need to establish the collection of outcomes data as a matter of routine. All of this has been moving forward in the context of the NHS quality agenda,^4^ which is underpinned by three themes: effective services, safety and positive patient experience.

Three main types of outcome measures have been proposed and are now seen as an absolute requirement within NHS services:

- clinician-rated outcome measures (CROMS)
- patient-rated outcome measures (PROMS)
- patient-rated experience measures (PREMS).

Various attempts have been made, particularly by the College's Faculty of Liaison Psychiatry, to reach a conclusion as to what measures should be recommended for use across all liaison psychiatry services. This has involved work at strategy days and in workshops at two annual residential conferences, with elements of this informing work subsequently carried out by colleagues at the Centre for Mental Health and culminating in a report.^3^ This report provided a clear and structured account of the challenges faced in attempting to measure outcomes consistently in liaison psychiatry. The difficulties particularly relate to the variety of liaison psychiatry service settings and types of intervention. These include in-reach work within general hospital emergency departments and/or medical and surgical in-patient wards, the provision of specialist out-patient services (generic or single-condition/service area), and in some cases designated liaison psychiatry in-patient beds. Within these various settings, contacts and interventions may include: single assessments, multiple assessments, diagnosis/formulation, guidance/advice, changes to current treatment, brief interventions, triage into and signposting of other services, longer-term psychotherapeutic and/or biopsychosocial interventions, and so on.

## The aim: developing a framework

Taking this complexity into account, a working group within the Faculty of Liaison Psychiatry undertook to extend the findings of the Centre for Mental Health report^3^ by creating a framework to enable routine outcome measurement across liaison psychiatry services, with the inclusion of specified measures for all services to use.

The key points from the Centre for Mental Health report were:

- outcome and performance measurement in liaison psychiatry services is at present very variable in content and quality
- liaison psychiatry services operate in a number of different settings and clinical environments, carrying out a wide range of different activities in support of patients with many different types of clinical problems
- most measurement frameworks for assessing quality and performance of services build on the ‘logic model’ developed in the 1960s, which focuses on the following three aspects:
  - structure – the key resources or inputs available in the settings concerned
  - process – what is actually done in the delivery of healthcare in terms of specific activities, with measurement based on quantifiable outputs such as the numbers of patients seen/treated
  - outcome – referring to any consequence of healthcare in terms of changes or benefits which result from the activities and outputs of the service in question.^5^

As also identified in the report:

- the optimal strategy for assessing quality and performance is to include a mix of indicators drawn from the three dimensions of structure, process and outcome: the so-called ‘balanced scorecard’ approach
- the complexity and heterogeneity of the service provision in liaison psychiatry necessarily rules out any (single) very simple, all-purpose approach to the measurement of the outcomes of performance in this context.

Accepting that no single instrument can be universally applied across the whole of liaison psychiatry, necessitating different groups of outcome measures (i.e. scorecards) for different contexts, the working group considered it essential to ensure that the approach is simple, easy to apply and consistently deliverable. In line with this aim, the FROM-LP has been developed and is proposed for adoption across all liaison psychiatry services in the NHS. Both the framework and this proposal have the full support of the Royal College of Psychiatrists' Faculty of Liaison Psychiatry.^1^ NHS devolution introduces some differences for patients and clinicians across the four countries of the UK, but well-organised outcome measurement is essential, whether services are in England, Scotland, Wales or Northern Ireland.

## The framework: the FROM-LP

The FROM-LP has been constructed in such a way as to enable consistency of data collection and the effective reporting of outcomes in individual liaison psychiatry services, thereby allowing the various ‘customers’ of liaison psychiatry (patients, carers, referrers and commissioners) to understand and have confidence in the beneficial effects of our services.

This initiative is being introduced at a critical time, when liaison psychiatry services need to move rapidly to a position of being able to provide meaningful data on relevant outcomes.

Improvements to the approach may come later, perhaps as a result of experience of using the framework, but there is a clear need to move forward with this as a matter of some urgency. To continue to discuss and attempt to find a ‘perfect’ approach before introducing anything would be unwise.

With reference to the logic model outlined above, the proposal is for structure (inputs) to be an issue for local services, informed in part by the Psychiatric Liaison Accreditation Network (PLAN), the College-based accreditation scheme which services are being encouraged to sign up to (www.rcpsych.ac.uk/workinpsychiatry.qualityimprovement/ccqiprojects/liaisonpsychiatry/plan/aspx).

The FROM-LP will focus on brief, simple and deliverable data collection regarding process and in particular outcomes. As noted above, it is important for outcome measurement to include elements covering clinician-rated clinical outcomes, patient-rated clinical outcomes and patient-rated satisfaction, but the FROM-LP also includes a fourth element: referrer-rated satisfaction. Given the broad range of clinicians and services which refer to liaison psychiatry, whether frequently or infrequently, the working group considered it important for liaison psychiatry services to be able to collect feedback from this particular category of their customers.

To keep the approach as straightforward as possible, the FROM-LP defines only two clinical case types, according to whether they involve a single clinical contact (case type 1) or a series of clinical contacts (case type 2) by the liaison psychiatry team. It is felt that the setting itself need not determine the measurement approach, rather the type of clinical contact. It is acknowledged that services may have some additional local data collection requirements, beyond those stipulated in the framework.

**Fig. 1:**
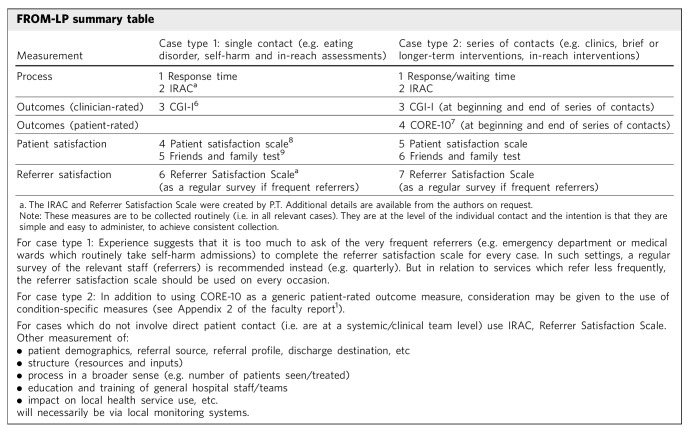
Framework for Routine Outcome Measurement in Liaison Psychiatry (FROM-LP) content. CGI, Clinical Global Impression – Improvement scale; CORE-10, Clinical Outcomes in Routine Evaluation (10-item version); IRAC, Identify and Rate the Aim of the Contact.

The FROM-LP summary table is presented in Fig. 1, and all outcome measurement requirements, as well as relevant tools and scales cited in the summary table, are available in the College's faculty report FR/LP/02 (Appendix 1 and 2).^1^

## Conclusion

The FROM-LP was published as a faculty report in May 2015, with the intention that it be adopted by liaison psychiatry services across the NHS. The initial response has been very encouraging, with numerous services having already implemented it. PLAN is considering its utility within their accreditation scheme, potentially by establishing standards to promote its widespread use as ‘best practice’, and it is also being used by the Royal College of Psychiatrists as a model to stimulate the development of outcome measurement frameworks across other College Faculties. In addition, the devolved Scottish Government is exploring the possibility of its application not only within the field of liaison psychiatry but across the whole of mental health, in an amended form.

Our initial aim was to provide an effective approach to enable liaison psychiatry services to demonstrate their clinical outcomes and effectiveness, and in so doing to further justify and support investment in this important and growing specialty. A broader utility appears to be emerging, and we are hopeful that the framework will have a wider impact over time.

## References

[1] Trigwell P Kustow J Santhouse A Gopinath R Aitken P Reid S *Framework for Routine Outcome Measurement in Liaison Psychiatry (FROM-LP)* (Faculty Report FR/LP/02). Royal College of Psychiatrists, 2015 (http://www.rcpsych.ac.uk/pdf/FRLP02.pdf).

[2] Parsonage M Fossey M Economic Evaluation of a Liaison Psychiatry Service. Centre for Mental Health, 2011.

[3] Fossey M Parsonage M Outcomes and Performance in Liaison Psychiatry: Developing a Measurement Framework. Centre for Mental Health, 2014.

[4] Department of Health The Operating Framework for the NHS in England 2012/13. Department of Health, 2011.

[5] Donabedian A Evaluating the quality of medical care. Milbank Mem Fund Q 1966; 44: 166–203. 5338568

[6] Guy W ECDEU Assessment Manual for Psychopharmacology. U.S. Department of Health, Education and Welfare, Public Health Service, Alcohol, Drug Abuse, and Mental Health Administration, National Institute of Mental Health, Psychopharmacology Research Branch, Division of Extramural Research Programs, 1976.

[7] Barkham M Bewick BM Mullin T Gilbody S Connell J Cahill J The CORE-10: A short measure of psychological distress for routine use in the psychological therapies. Counselling Psychother Res 2013; 13: 3–13.

[8] Persaud A Pearson S Oates M Strathdee G Aguis M Higgit A Mental Health Outcomes Compendium. National Institute for Mental Health in England,2008.

[9] Department of Health The NHS Friends and Family Test Implementation Guidance. Department of Health, 2012.

